# Role of Fra-2 in cancer

**DOI:** 10.1038/s41418-023-01248-4

**Published:** 2023-12-16

**Authors:** Gian Luca Rampioni Vinciguerra, Marina Capece, Giorgia Scafetta, Sydney Rentsch, Andrea Vecchione, Francesca Lovat, Carlo M. Croce

**Affiliations:** 1 Department of Cancer Biology and Genetics and Comprehensive Cancer Center, The Ohio State University, Columbus, OH USA; 2 Department of Clinical and Molecular Medicine, Faculty of Medicine and Psychology, Sant’Andrea Hospital, University of Rome “Sapienza”, Rome, 00189 Italy

**Keywords:** Cancer genomics, Cancer microenvironment

## Abstract

Fos-related antigen-2 (Fra-2) is the most recently discovered member of the Fos family and, by dimerizing with Jun proteins, forms the activator protein 1 (AP-1) transcription factor. By inducing or repressing the transcription of several target genes, Fra-2 is critically involved in the modulation of cell response to a variety of extracellular stimuli, stressors and intracellular changes. In physiological conditions, Fra-2 has been found to be ubiquitously expressed in human cells, regulating differentiation and homeostasis of bone, muscle, nervous, lymphoid and other tissues. While other AP-1 members, like Jun and Fos, are well characterized, studies of Fra-2 functions in cancer are still at an early stage. Due to the lack of a trans-activating domain, which is present in other Fos proteins, it has been suggested that Fra-2 might inhibit cell transformation, eventually exerting an anti-tumor effect. In human malignancies, however, Fra-2 activity is enhanced (or induced) by dysregulation of microRNAs, oncogenes and extracellular signaling, suggesting a multifaceted role. Therefore, Fra-2 can promote or prevent transformation, proliferation, migration, epithelial-mesenchymal transition, drug resistance and metastasis formation in a tumor- and context-dependent manner. Intriguingly, recent data reports that Fra-2 is also expressed in cancer associated cells, contributing to the intricate crosstalk between neoplastic and non-neoplastic cells, that leads to the evolution and remodeling of the tumor microenvironment. In this review we summarize three decades of research on Fra-2, focusing on its oncogenic and anti-oncogenic effects in tumor progression and dissemination.

## Facts

- Fra-2 is a member of Fos transcription factor family, involved in several cellular functions.
- As result of microRNAs dysregulation and oncogene activation, Fra-2 is frequently overexpressed in human malignancies.
- Fra-2 transcriptionally modulates several target genes, mediating growth, epithelial-mesenchymal transition, invasion, metastatic dissemination and drug resistance of cancer cells.
- Fra-2 expression in neoplastic cells and tumor-associated cells promotes microenvironment remodeling and immune escape.

## Open questions

- What genes are regulated by Fra-2 during tumor progression?
- How does Fra-2 remodel the tumor microenvironment?
- Does Fra-2 targeting represent a valuable approach in therapy?

## Introduction

First identified in 1990, Fos-related antigen-2 (hereafter, Fra-2) is an inducible transcription factor that belongs to the Fos protein family together with c-Fos, FosB and Fra-1 [1].

Like the other Fos proteins, Fra-2 exerts its function by heterodimerizing with Jun family members, forming a complex called activator protein 1 (AP-1) [2, 3].

**Fig. 1:**
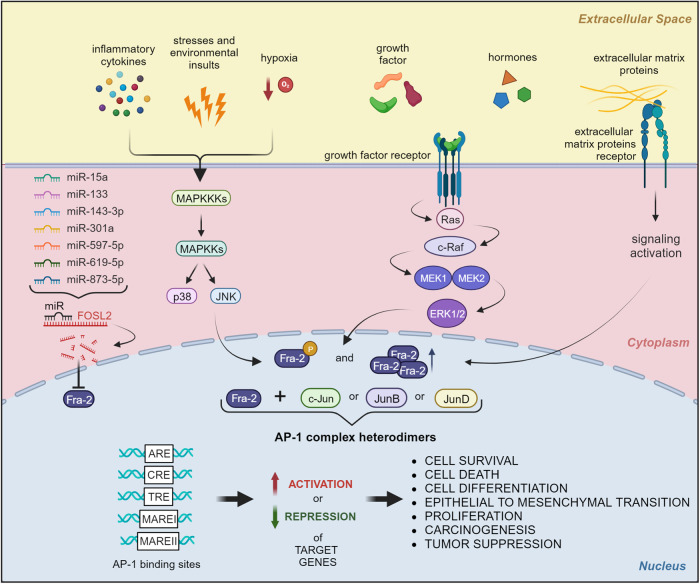
Fos-related antigen-2 (Fra-2) is modulated by microRNAs, extracellular signals, environmental stresses, and oncogenes. MicroRNAs regulate translational efficiency of FOSL2 mRNA, inhibiting its expression. When phosphorylated or activated, Fra-2 forms AP-1 complex by dimerizing with Jun family members. AP-1 heterodimers recognize specific genomic response-elements (ARE, CRE, TRE, MARE I and MARE II) and transcriptionally control, activating or repressing, a wide number of genes. Modulation of those genes contributes to cell survival, cell death, cell proliferation, epithelial-mesenchymal transition, tumor progression and carcinogenesis. Created with BioRender.

Despite the evidence accumulated in the past three decades, a comprehensive understanding of the role of Fra-2 in physiologic and neoplastic contexts is still needed. This task is complicated by the variety of modulators and interactors that mediate Fra-2 activity (Fig. 1).

Several microRNAs have been reported to target FOSL2, the gene encoding Fra-2, at post-transcriptional level. For instance, dysregulation of miR-143-3p, miR-133, miR-301a and miR-597-5p eventually contributes to Fra-2 overexpression in osteosarcoma [4], hepatocellular carcinoma [5], non-small cell lung cancer [6] and colorectal carcinoma [7], respectively.

Fra-2 can be rapidly induced by a series of different stimuli including hormones, serum growth factors, cytokines, cell–matrix interactions, and a plethora of stresses and environmental insults [1, 2, 8–12]. In 3T3 fibroblasts, serum stimulation induces post-translational modification of Fra-2 during G0/G1, but not S/M phase, suggesting that Fra-2 activity can also be influenced by cell cycle progression [13]. Inflammatory cytokines and cell stress induce Fra-2 activation *via* JNK and p38MAPK pathways, whereas serum and growth factors stimulation are mainly mediated by ERK, that directly phosphorylates Fra-2, enhancing its DNA binding capabilities [2, 12]. Other kinases (such as PKA, PKC and cdc2) may virtually phosphorylate Fra-2 [13, 14].

Once activated, Fra-2 interacts with Jun proteins, forming different combinations of heterodimers that further contribute to diversifying the transcriptional activity of Fra-2 [15]. In addition, Fra-2-containing dimers recognize different DNA-binding sites and their interaction may result in increased or reduced transcription of target genes [2].

Consequently, Fra-2 drives a diverse collection of contrasting biological mechanisms, spanning from cell survival and death, differentiation and proliferation, carcinogenesis, and tumor suppression [15].

Focusing on cancer, Fra-2 overexpression shows transforming properties in chicken embryo fibroblasts (CEF), but not in rat fibroblasts [1, 16]. As discussed below, Fra-2 activity favors metastasis dissemination in breast [17] and colorectal cancer [7], whereas it prevents migration and invasion in malignant melanoma [18].

Despite Fos family members sharing a certain grade of similarity, several studies have clarified that each Fos protein has distinct features and plays different functions in cancer.

In fact, Fra-2, as well as Fra-1, lacks the C-terminal transactivating domain present in c-Fos and FosB proteins, displaying a weaker transforming capability compared to c-Fos in rodent fibroblasts [1, 16]. Moreover, Fra-2/c-Jun and c-Fos/c-Jun dimers differentially regulate gene transcription in mouse embryonic carcinoma cell lines [19].

In 3T3 murine fibroblasts, Fra-2 was found to be the main partner of c-Jun [20, 21]. In this context, the overexpression of Fra-2/c-Jun dimers, but not c-Fos/c-Jun, sustains proliferation of serum starved cells by upregulating Cyclin-D1 and Cyclin-A2^3^. Furthermore, Fra-2 induces transcription of Relb, a subunit of NF-kB, whereas c-Fos does not [22].

In CSML0 murine neoplastic cells, ectopic expression of Fos proteins promotes the transcription of different genes. c-Fos and Fra-1 activate genes of the urokinase system, whereas Fra-2 controls thrombospondin-1, osteopontin and CD44 levels [23].

Studies conducted on transgenic mouse models show that, in contrast to c-Fos [15], ectopic expression of Fra-2 fails in promoting tumorigenesis, whereas affects eye development or elicits a pro-fibrotic phenotype [24–28]. Consistently, a recent work on a small cohort of patients reported that germinal mutations in FOSL2 have been associated with neurodevelopmental delay and no increased risk of cancer was observed [29].

Those results suggest that Fra-2 overexpression alone is not sufficient to activate carcinogenesis, but it may likely act as a mediator of other oncoproteins.

It is known that some oncogenes like Src, Ras and Raf require AP-1 expression to exert their pro-tumorigenic activity, while other oncogenes like Ros and Myc do not [30]. In this context, Src, H-Ras, K-Ras and Raf oncogenes strongly induce upregulation and phosphorylation of Fra-2, which seems essential for cell transformation [21, 30, 31].

**Fig. 2:**
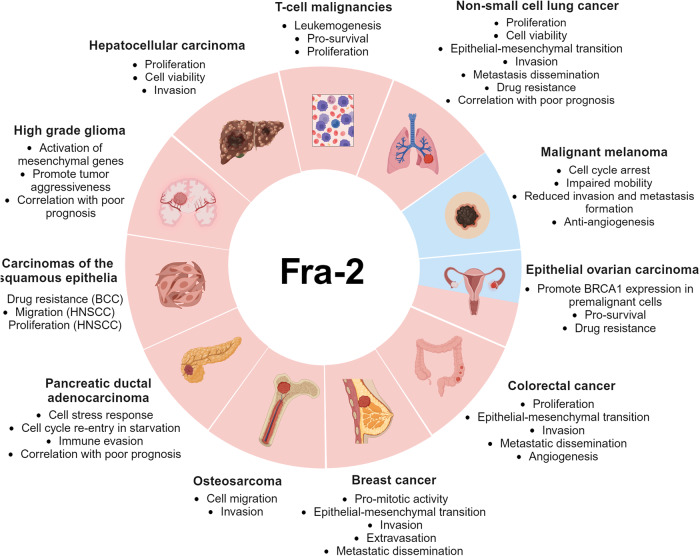
Fra-2 plays a critical role in cancer. Fra-2 can exert a pro-tumorigenic behavior (labeled in red) in tissues and organs such as liver, lung, breast, and pancreas or can play an oncosuppressive role (labeled in blue) in malignant melanoma, or a dual role as in epithelial ovarian carcinoma. Created with BioRender.

Altogether, these results point to Fra-2 as an important player in cancer. In this review, we summarize the mediatory role of Fra-2 at the intricate crossroad between microRNAs dysregulation, oncogenes expression, extracellular signaling pathways and cell stress response in tumor progression and dissemination (Fig. 2 and Table 1).

**Table 1:** Fra-2-mediated activities in human malignancies.

Cancer type	Role of Fra-2 in cancer	Fra-2 target gene	References
Hepatocellular Carcinoma	proliferation cell viability invasion	–	[5]
Osteosarcoma	cell migration invasion	*–*	[4]
Breast Cancer	pro-mitotic activity EMT invasion extravasation metastatic dissemination	*RELB* *ICAM-1* *L1-CAM*	[50–52]
Non-Small Cell Lung Cancer	proliferation cell viability EMT invasion metastasis dissemination drug resistance	*SNAI2* *CASC9* *GLIPR1*	[6, 39, 59, 60, 62]
Malignant Melanoma	cell cycle arrest impaired mobility reduced invasion reduced metastasis formation anti-angiogenesis dedifferentiation	*FAM212B*	[18, 67]
Pancreatic Ductal Adenocarcinoma	cell stress response cell cycle re-entry in starvation immune evasion	*IGF1R*	[10, 73, 75]
Colon Cancer	proliferation EMT invasion metastasis dissemination angiogenesis	*E-CADHERIN* *VIMENTIN* *FIBRONECTIN* *EPHA2*	[7, 77, 79, 80]
Carcinomas of the Squamous Epithelia	drug resistance (BCC) migration (HNSCC) proliferation (HNSCC)	*–*	[84, 86]
Epithelial Ovarian Carcinoma	pro-survival drug resistance	*BRCA1*	[91, 93]
High Grade Gliomas	promote tumor aggressiveness	MGES* NES** *ANXA1*	[94, 95]
T-Cell Malignancies	leukemogenesis pro-survival proliferation	*C-MYB* *MDM2* *BCL6* *SOX4* *CCR4*	[104, 105, 106, 107]

**-** not known

*EMT* Epithelial-Mesenchymal Transition, *BCC* Cutaneous Basal Cell Carcinoma, *HNSCC* Head and Neck Squamous Cell Carcinomas, *MGES* Mesenchymal GeneExpression Signature, *NES* Natural Evolution Signature.

Table summarizes the role of Fra-2 in different cancer types and the validated targeted genes.

### Fra-2 and the TGFβ pathway

As previously discussed, Fra-2 transgenic mice display a pro-fibrotic phenotype characterized by a dramatic activation of fibroblasts and macrophages, eventually promoting systemic sclerosis, lung fibrosis, vascular remodeling, and inflammation [25–27, 32, 33]. These studies have contributed to highlighting the close interaction between Fra-2 and the transforming growth factor-b (TGFβ) signaling.

The TGFβ cytokine is a crucial regulator of the differentiation of fibroblasts in activated-fibroblasts or myofibroblasts, by promoting the expression of myogenic proteins and extracellular matrix (ECM) components [34]. The canonical TGFβ signaling pathway is mediated by Smad transcription factors, while a non-canonical pathway engages ERK 1/2 proteins [9]. Accumulated evidence suggests that Fra-2 is involved in the TGFβ pathway both upstream and downstream, by transcriptionally inducing TGFB1 gene expression and operating as an effector of canonical and non-canonical TGFβ signaling cascades [9, 28, 32, 35].

Under pro-inflammatory stimuli, Fra-2/c-Jun dimers drive TGFB1 gene transcription in IL-13-activated monocytes-macrophages, promoting lung fibrosis and colitis in mice [28]. In cardiac fibroblasts, Fra-2 transcriptionally upregulates TGFβ expression, contributing to post-infarction fibrosis and heart remodeling [11]. In fibroblasts from patients with systemic sclerosis, TGFβ and PDGF administration activates Fra-2 expression *via* non-canonical ERK signaling that, in turn, increases collagen deposition in the ECM [32].

In bovine endothelial cells, Fra-2/JunB dimers are activated by canonical and non-canonical TGFβ signaling, leading to the expression of LOXL4, a secretory enzyme involved in vascular ECM assembly [36]. In addition, Fra-2/JunB dimers are responsible for transcriptional upregulation of meprin-β, a TGFβ-induced metalloproteinase that mediates vascular remodeling in pulmonary hypertension [37]. Consistent with these studies [36, 37], TGFβ inhibition significantly attenuates the vascular alterations in a Fra-2 transgenic model of systemic sclerosis [27].

In cancer, the TGFβ pathway is primary involved in the tumor evolution, mainly by promoting the epithelial-mesenchymal transition (EMT) [34, 38], a mechanism on which Fra-2 overexpression also converges [7, 39].

In some tumors like non-small cell lung cancer (NSCLC) and hepatocellular carcinoma (HCC), the direct interaction between Fra-2 activity and TGFβ signaling has been demonstrated [5, 40].

Indeed, TGFβ1 stimulation induces Fra-2 upregulation that, in turn, directly interacts with Smad3, promoting cell migration and EMT of NSCLC cells. Consistently, Fra-2 and phosphorylated Smad3 expression show a significant correlation with postoperative relapse and shorter survival in NSCLC patients [40].

In HCC cells, TGFβ administration stimulates the expression of Fra-2 and Smad3, that sustain cell viability, proliferation, migration and invasion [5]. In this model, the ectopic expression of miR-133a counteracts the TGFβ signaling by Fra-2 targeting [5].

In primary human breast fibroblasts, TGFβ signaling induces Fra-2 expression *via* ERK that, in turn, activates the transcription of TGFβ targeted genes like Fibronectin and α-SMA [9]. This mechanism has a prominent role in breast cancer (BC), where myofibroblasts contribute to tumor microenvironment (TME) reshaping and tumor progression. Lately, tamoxifen has been described also as an anti-fibrotic mediator in some fibrotic diseases, inhibiting the activation of TGF-β-stimulated fibroblasts. Therefore we can speculate that, the administration of tamoxifen, a selective estrogen receptor (ER) modulator used in the treatment of ER-positive BC, could inhibit Fra-2 activation in tumor associated fibroblasts, eventually exerting an anti-fibrotic property on the TME [9]. To date, there are no evidence of the role and function of Fra-2 in cancer-associated fibroblasts. Since tamoxifen can decrease mRNA transcript of Fra-1 in MCF-7 cells [41], we could hypothesize that the modulation of genes in cancer-associated fibroblast might be coordinately achieved by tamoxifen-induced Fra-1 and Fra-2 inhibition, involving differences in AP-1 complex motif recognition by Fra-1 and Fra-2 [42].

### Role of Fra-2 in osteosarcoma

As discussed, Fra-2 transcriptionally mediates many activities of fibroblasts. Accumulated evidence indicates that, under physiologic conditions, Fra-2 is also markedly expressed in other mesenchymal-derived cells, contributing to the differentiation and homeostasis of bones [43], muscles [44], cartilages [45], and adipose tissue [46].

In bones, Fra-2 was found to promote the apoptosis of osteoclasts *via* Bcl2 downregulation. Consequently, Fra-2-deficient mice display giant osteoclasts and reduced mineralization of bones [43].

Moreover, Fra-2 controls the expression of collagen1α2 and osteocalcin, respectively promoting deposition of osteoid ECM and the endocrine functions of osteoblasts [47, 48]. Since osteosarcomas of osteoblastic and chondroblastic origin retain the expression of Fra-2 and its molecular targets, their application has been proposed for the differential diagnosis of bone-derived tumors [47].

In osteosarcoma, the oncosuppressor miR-143-3p directly targets Fra-2. Then, the downmodulation of miR-143-3p unleashes Fra-2 expression that, in turn, increases migration and invasion of osteosarcoma cell lines [4].

Altogether, these findings reinforce the prominent role of Fra-2 in mesenchymal cells for both physiologic and neoplastic contexts.

### Role of Fra-2 in breast cancer

Breast cancer (BC) is the most common cancer in women worldwide. Based on the molecular status of estrogen receptor (ER), progesterone receptor (PgR) and human epidermal growth factor-2 (HER2), BC is categorized into 3 major subtypes: hormone receptor positive, HER2 positive, and triple-negative tumors, which lack all 3 molecular markers [49]. This classification deeply influences the prognosis: indeed, hormone receptor positive BC generally has a more favorable outcome, whereas triple-negative BC is more aggressive, and few therapeutic alternatives exist to treat it [49]. To date, metastatic disease is reported in 6% of cases at the time of diagnosis [49]. Since virtually all BC-related deaths are due to metastatic dissemination, many efforts have been made to understand the molecular mechanisms that drive cell migration and dissemination.

**Fig. 3:**
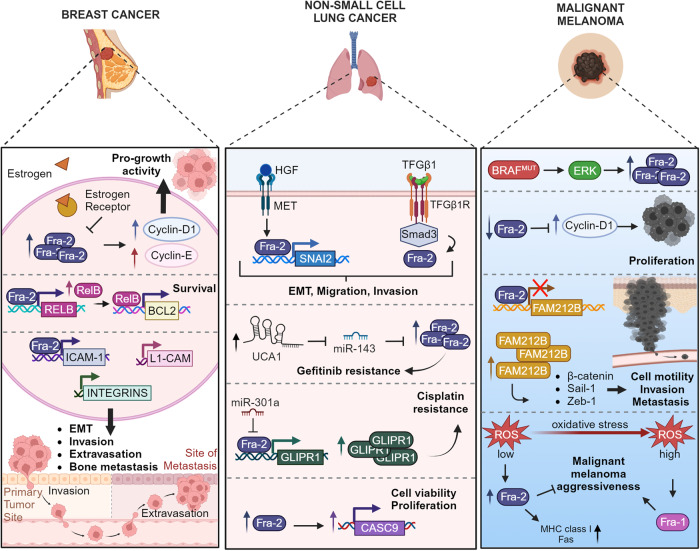
Role of Fra-2 regulation in breast (BC), non-small cell lung cancer (NSCLC) and malignant melanoma (MM). In BC, Fra-2 is modulated by ERa and, in turn, transcriptionally activates Cyclin-D1 and -E, supporting pro-growth activity and Relb, promoting cell survival. Fra-2 stimulates epithelial-mesenchymal transition (EMT) and extravasation inducing transcription of ICAM-1, L1-CAM and Integrins. In NSCLC, MET and TGFβ1 signaling induce Fra-2 activation that, in turn, transcriptionally controls SNAI2 and interaction with Smad3, respectively. These extracellular signals promote EMT and cell motility *via* Fra-2. Moreover, Fra-2 contributes to cell proliferation, acting on cancer susceptibility candidate 9 (CASC9). Fra-2 is also involved in the mechanism of drug resistance: in gefitinib-resistant NSCLC cells upregulation of exosomal lncRNA urothelial carcinoma-associated 1 (UCA1) induces Fra-2 expression. Moreover, in miR-301a-low context, Fra-2 promotes platinum resistance, modulating GLIPR1. In MM, Fra-2 exerts a oncosuppressive role, in fact Fra-2 is downmodulated in human melanoma samples compared to normal skin. Fra-2 low- expression induces Fam212b and in turn promotes cell motility and metastasis and inhibits CyclinD1, controlling cell proliferation. Reduced levels of ROS cause Fra-2 overexpression, leading to a less aggressive phenotype. Created with BioRender.

Fra-2 is abundantly expressed in tissue samples of BC (Fig. 3). In patients, Fra-2 expression significantly correlates with nodal involvement, younger age at diagnosis and early relapse, whereas high c-Fos expression is associated with a favorable outcome [50, 51].

Accumulated evidence indicates that ER signaling reduces Fra-2 expression in BC cells. Thus, the pharmacological inhibition of ER restores Fra-2 levels [52], suggesting that Fra-2 may contribute to hormone therapy response in ER-positive BC.

Conversely, Fra-2 is overexpressed in ER-negative BC cell lines and transcriptionally activates the NF-kB subunit Relb, promoting cell survival *via* Bcl2, EMT and bone metastasis [52].

Moreover, Fra-2 represses transcription of cell-cell contact mediating genes and promotes expression of adhesion molecules involved in extravasation (e.g., ICAM-1, L1-CAM and integrins). Consistently, Fra-2, ICAM-1 and L1-CAM levels show a direct correlation in a clinical cohort of BC [51].

Thus, Fra-2-overexpressing BC cells show a higher capability to adhere to ECM components, to interact with glycoproteins on the vascular endothelial surface and to form metastases in xenograft mouse models [51, 53].

Although Fra-2 overexpression does not accelerate proliferation rate of BC cells in vitro [17], Fra-2 expression significantly correlates with Cyclin-D1 and Cyclin-E levels in BC samples [54], suggesting that Fra-2 could also exert a pro-growth activity.

Collectively, these results largely support the fact that Fra-2 expression is more associated with hormone receptor negative tumors and fosters the tumor progression and aggressiveness of BC.

### Role of Fra-2 in non-small cell lung cancer

Lung cancer is one of the most common cancers and is the leading cause of tumor-related deaths worldwide [55]. From a histological point of view, non-small cell lung cancer (NSCLC) accounts for 85% of cases, and adenocarcinoma and squamous cell carcinoma represent the main subtypes [55].

NSCLC harbors several driver mutations in oncogenes like RAS, EGFR, BRAF, ALK, ROS1 and MET that frequently lead to the hyperactivation of MAPKs [55, 56]. Since, as previously mentioned, AP-1 proteins are direct effectors of MAPK signaling [57], it is not surprising that they exert a critical function in NSCLC.

In normal lung epithelial cells, exposure to cigarette smoke and asbestos, which represent the primary carcinogens in the lung, determines upregulation and activation of c-Fos and Fra-1 *via* MAPK/JNK signaling, whereas Fra-2 levels remain substantially unchanged [58–60]. These findings suggest that Fra-2 may exert a marginal role in the induction of lung neoplasms.

By contrast, many observations support a deep involvement of Fra-2 in the later stages of NSCLC progression (Fig. 3). Indeed, Fra-2 was found to be highly expressed in NSCLC samples and significantly correlated with poor prognosis in adenocarcinoma patients [57]. In a genetic mouse model of NSCLC, Fra-2 inactivation strongly impairs tumor formation [57].

Another study identifies Fra-2 as a mediator of the MET receptor tyrosine kinase [39]. MET-induced phosphorylation and activation of Fra-2 enhance the transcriptional expression of SNAI2, a key regulator of EMT, eventually promoting invasion and migration in NSCLC cells [39].

It has been reported that Fra-2 is a predicted transcription factor of cancer susceptibility candidate 9 (CASC9), an oncogenic lncRNA that further contributes to cell viability and proliferation of squamous NSCLC cells [61].

Importantly, Fra-2 is also involved in the mechanisms of drug response; in particular, Fra-2 overexpression has been demonstrated in NSCLC cells and patients’ tissues with acquired resistance to the EGFR tyrosine kinase inhibitor gefitinib [62]. Mechanistically, the upregulated lncRNA urothelial carcinoma-associated 1 (UCA1) acts as a sponge of miR-143, eventually promoting Fra-2 expression and drug resistance [62]. Recently, our group identified a new role of Fra-2 in the response to cisplatin in NSCLC [6]. We discovered the novel miR-301a/Fra-2/GLIPR1 axis in which miR-301a targets Fra-2 and GLIPR1 and inversely correlates with their expression in NSCLC patients. In turn, Fra-2 promotes the transcription of GLIPR1, a mediator of cisplatin resistance [63]. In miR-301a-low context, Fra-2 contributes to platinum resistance *via* modulation of GLIPR1 [6], whereas administration of the AP-1 inhibitor T-5224 restores platinum sensitivity.

These findings confirm that, at least in an in vitro setting, the use of AP-1 inhibitors represents a valuable strategy in cancer [64, 65] and corroborate the possibility that Fra-2 is amenable for targeted therapy approaches [6, 66].

### Role of Fra-2 in malignant melanoma

As aforementioned, AP-1 is an important mediator of MAPK signaling [30]. Melanomagenesis is frequently driven by mutations in BRAF or NRAS oncogenes that lead to constitutive activation of MAPK cascade, eventually inducing both Fra-1 and Fra-2 expression in malignant melanoma (MM) cells [18, 67]. While Fra-1 drives transformation and EMT transdifferentiation of melanocytes [68], Fra-2 plays an oncosuppressive role in MM (Fig. 3).

Indeed, human melanoma samples display lower levels of Fra-2 compared to normal skin or nevus tissues. In patients, Fra-2 downmodulation correlates with clinical aggressiveness, metastatic disease and poor prognosis [18]. Consistently, Fra-2 silencing promotes cell proliferation *via* Cyclin-D1 upregulation in vitro, while increases neo-angiogenesis and metastasis formation in vivo. Mechanistically, Fra-2 binds and represses the promoter of Fam212b, encoding a protein involved in multiple actin-driven processes, that, in turn, drives cell motility and invasion by inducing β-catenin signaling, Snail-1 and other effectors [18].

Exposure to reactive oxidative species (ROS) can alter the composition of AP-1 dimers in MM cells [67]. Under high levels of oxidative stress, Fra-1-containing dimers are predominantly represented. However, when ROS levels are decreased by Resveratrol administration, AP-1 composition shifts from Fra-1 to Fra-2-containing dimers, that limits the aggressiveness of MM cells [67]. In fact, Resveratrol-induced Fra-2 overexpression exerts a suppressive role on AP-1 transcriptional activity in MM cells and is associated with cell growth impairment and increased expression of MHC class I antigen and Fas [67].

A recent study, focusing on the AP-1 involvement in melanoma cell plasticity, confirms that Fra-2 knockdown induces the expression of Fra-1 in melanoma cells. However, Fra-2 silencing is also followed by increased levels of SOX10, a marker of melanocytic differentiation, suggesting that Fra-2 expression may promote less differentiated states in melanoma cells [69]. These results appear to be in contrast with the data published before and add another level of complexity to the Fra-2 role in malignant melanoma, that certainly requires to be better clarified in future investigations.

### Role of Fra-2 in pancreatic ductal adenocarcinoma

In the last decade, pancreatic ductal adenocarcinoma (PDAC) has shown an increasing incidence and it is expected to become the second leading cause of cancer-related death in the future [70]. Nearly all PDAC harbors activating mutation of KRAS (KRAS^MUT^) that represents an early step in the transformation of pancreatic epithelium [70]. As previously discussed, activation of AP-1 proteins largely mediates KRAS-driven carcinogenesis [30]. Indeed, the trans differentiation of the normal epithelium into duct-like progenitor cells in chronic pancreatitis is mediated by Fra-1/JunB dimers; then, the insurgence of KRAS^MUT^ stabilizes AP-1 activation, locking the epithelium in a progenitor state, preceding the tumor initiation [71].

An outstanding feature of PDAC is the extraordinary capability to thrive and progress in a challenging TME, where fibroblasts activation and fibrotic stroma compress neoplastic cells and blood vessels, limiting the amount of oxygen and nutrients [72].

AP-1 proteins greatly contribute to the PDAC stress response to the TME [73, 74]. In vitro, PDAC cells respond to mechanical compression by activating MAPK/JNK signaling cascade that culminates in AP-1 recruitment [73]. Then, c-Jun drives a complex adaptive mechanism involving cell cycle arrest, activation of autophagy, cytoskeleton remodeling and cell migration [73].

**Fig. 4:**
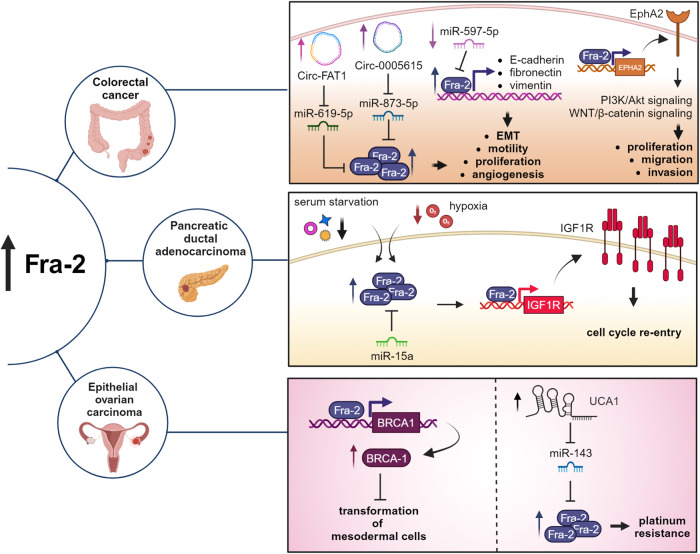
Role of Fra-2 in colon rectal cancer (CRC), pancreatic ductal adenocarcinoma (PDAC) and epithelial ovarian carcinoma (EOC). In CRC, different non-coding RNAs regulate Fra-2 expression. In particular, miR-597-5p low expression is not capable to target Fra-2 that in turn transcriptionally activates E-cadherin, Fibronectin and Vimentin, promoting EMT, cell motility and neo-angiogenesis. Circ-FAT-1 and Cir-0005615 overexpression induce Fra-2 transcriptional activity, sponging miR-619-5p and miR-873-5p, respectively. Moreover, Fra-2 activates the expression of EphA2, turning on PI3K/Akt and WNT/β-catenin signaling pathways. In PDAC, Fra-2 expression is modulated by miR-15a and different stressors and, in turn, induces IGF1R upregulation, promoting cell cycle re-entry in serum starved cells. In EOC, Fra-2 regulates BRCA1 expression, protecting mesodermal cells from the transformation in early stage of carcinogenesis. Conversely, LncRNA-UCA1 upregulation, sponging miR-143, induces Fra-2 overexpression and confers platinum resistance in advanced disease. Created with BioRender.

Analysis of PDAC cell proteome reveals that Fra-2 expression is potently induced by hypoxia and starvation [10]. Our group has recently shown that the tumor suppressor miR-15a impairs PDAC response to serum restriction by targeting Fra-2 expression. In miR-15a low expressing cells, serum starvation induces Fra-2 upregulation that, in turn, leads to IGF1 receptor (IGF1R) overexpression, mTOR pathway activation and cell cycle re-entry (Fig. 4) [75].

Noteworthy, recent single-cell multiomic analyses showed that Fra-2 levels are significantly higher in PDAC compared to normal epithelial cells and strongly correlate with shorter survival of patients [76]. The study of genomic regions with open chromatin features reveals a significant enrichment of Fra-2 binding motif in PDAC compared to normal epithelial cells [76].

These intriguing results support the idea that Fra-2 is a crucial transcription factor in PDAC, encouraging future investigations in this field.

### Role of Fra-2 in colorectal cancer

Colorectal tumorigenesis is a multistep process involving genetic alterations of oncogenes and tumor suppressor genes, which leads to transformation of normal epithelium in adenoma and eventually in colorectal cancer (CRC). Mutation of the adenomatous polyposis coli (APC) tumor suppressor gene occurs in early carcinogenesis, leading to dysregulation of several downstream targets, including AP-1 [77]. As previously observed in lung and pancreatic tumorigenesis [58, 71], also in CRC the AP-1 proteins show different expression profiles, suggesting different contributions in tumor initiation and progression.

Indeed, immunohistochemical analysis of a cohort of adjacent normal mucosa and colorectal lesions at different stages of transformation, revealed that Fra-2 is weakly positive in a small percentage of epithelial cells in the normal mucosa, positively expressed in 26% of adenomas and strongly expressed in all CRC samples. By contrast, c-Fos is strongly expressed in both normal and colorectal lesions, whereas Fra-1 expression is only detectable in adenomas and CRC. These results lead to the conclusion that Fra-2 overexpression is not a frequent event in benign adenomas, raising the possibility that Fra-2 might contribute mainly to tumor progression during colorectal tumorigenesis [77] (Fig. 4).

Expression of Fra-2 has been demonstrated to be regulated also by miR-597-5p in CRC [7] and BC [78]. In paired samples of CRC and adjacent normal mucosa, Fra-2 expression is higher in neoplastic cells than in normal epithelium and inversely correlates with miR-597-5p levels [7]. Ectopic overexpression of miR-597-5p in CRC cell lines significantly reduces Fra-2 expression and the metastatic dissemination of CRC cells in mice. Mechanistically, Fra-2 expression promotes EMT, reducing E-Cadherin expression and upregulating Vimentin and Fibronectin [7].

Other studies point to miR-619-5p and miR-873-5p targeting of Fra-2 as a regulatory mechanism in CRC [79, 80]. According to the Authors, the upregulation of Circ-FAT1 or Circ-0005615 promotes Fra-2 expression in CRC cells, acting as a sponge for miR-619-5p and miR-873-5p, respectively. Therefore, circ-FAT1 increases motility, proliferation and angiogenesis of neoplastic cells *via* Fra-2 [79, 80].

Fra-2 has been demonstrated to be a critical member of the EphA2-super enhancer (EphA2-SE) clusters, a group of transcription factors that drive tumorigenesis by activating the expression of EphA2 receptor [81]. Therefore, in different models, including BC and CRC cell lines, deletion of EphA2-SE impairs proliferation, migration and invasion by suppressing EphA2 downstream pathways like PI3K/Akt and WNT/β-catenin signaling [81].

### Role of Fra-2 in the carcinomas of the squamous epithelia

In the epidermis, Fra-2 plays opposite roles compared to c-Fos [14]. c-Fos increases the transcription of AP-1 target genes during the differentiation of squamous epithelial cells, whereas Fra-2 is eminently expressed in the early basal layer and in the late granular layer, where it exerts a repressive role on AP-1 transcriptional activity [82]. Therefore, it could be reasonable to think that Fra-2 may contribute to down-regulating genes not relevant for the late stages of epidermal differentiation [14].

However, Fra-2 expression is also important for the neoplastic transformation of the skin. In the different steps of mouse skin carcinogenesis, AP-1 members’ expression and activity change. In particular, high amount of Fra-1 phosphorylation, mediated by Ras and JNK, is found in early stages of tumorigenesis, whereas c-Jun and Fra-2 expression promotes proliferation and invasion in the late stages of carcinogenesis, possibly via Cyclin-D1 and metalloproteases [83]. As previously described in NSCLC, PDAC and CRC, Fra-2 seems to be more involved in the late phases of tumor progression.

Cutaneous basal cell carcinoma (BCC) is the most common cancer in humans, specifically driven by overactivation of the Hedgehog (Hh) signaling. Deregulation of Hh pathway results in activation of glioma-associated oncogene (Gli) transcription factors that, in turn, lead to proliferation and transformation of keratinocytes [84]. Consequently, targeted therapies against Hh pathway (SMO inhibitors) are promising candidates for the treatment of BCC. However, mechanisms of non-canonical activation of Gli1 represents the major limit for the success of this therapeutic strategy.

Fra-2 is the only Fos family members found to be overexpressed in BCC cells [66]. In this context, JNK-mediated Fra-2 activation induces a signaling cascade resulting in Gli1 stimulation by non-canonical Hh pathway and SMO-inhibitors resistance. Treatment of BCC with the AP-1 inhibitor T5524 downmodulates Gli1 expression and inhibits cell viability. Altogether, these results point to AP-1 as a critical driver of drug resistance in BCC and to Fra-2 as the main contributor of this phenotype among the other Fos proteins [66]. As previously described in NSCLC [6], combination of AP-1 inhibitors with other drugs might revert the oncogenic phenotype, sensitizing cancer cells to apoptotic process.

Head and neck squamous cell carcinomas (HNSCC) represent a clinical heterogeneous group of malignancies of the oral cavity, pharynx and larynx, frequently diagnosed in advanced stage of disease [85]. The infection of HPV (human papillomavirus), particularly HPV-16 and HPV-18, is an independent risk factor, responsible for a substantial proportion of HNSCC [86].

In HNSCC of the tongue, AP-1 DNA binding activity is significantly increased in tumor samples compared to the normal adjacent tissue, and c-Jun along with c-Fos and Fra-2 are the principal components of AP-1 dimers [86]. Thus, Fra-2 silencing is accompanied by upregulation of p53 and concomitant downmodulation of c-Fos, c-Jun and the viral oncoproteins E6/E7 in HPV + HNSCC cells.

Abolition of Fra-2 also reduces expression of AP-1 target genes, such as MMP9 and Cyclin-D1, inhibiting migration and cell cycle progression of neoplastic cells [86].

Altogether, these results demonstrate the tumorigenic role of Fra-2 in HNSCC of the tongue and support the possibility that upregulation of p53 *via* Fra-2 inhibition could sensitize tumors to the treatments [86].

### Role of Fra-2 in epithelial ovarian carcinoma

Epithelial ovarian carcinoma (EOC) is a rare (2.5% of all cancers in women) but highly lethal disease, representing the leading cause of death due to gynecologic malignancy worldwide [87, 88]. The high lethality of EOC is due to the recalcitrant chemoresistance and the highly metastatic potential of neoplastic cells. Indeed, EOC cells can disseminate to the peritoneal walls and organs by freely floating in the abdominopelvic cavity [89]. Intriguingly, the lack of interactions with ECM substrata in floating premalignant cells has been shown to impact on the expression of BRCA1, a gene frequently downmodulated in sporadic EOC and controlled by Fra-2 by binding CRE domain in the promoter [90]. When cultured in suspension, premalignant mesodermal cells show a drastic reduction in Fra-2 activity along with BRCA1 expression. Mechanistically, suspension-mediated downmodulation of Fra-2 contributes to an initial decrease of BRCA1 expression that could prelude to a stable suppression by methylation of BRCA1 promoter, as typically observed in advanced EOC (Fig. 4) [90].

According to these data, Fra-2 could exert a protective role in the early phases of carcinogenesis of mesodermal cells. By contrast, Fra-2 has been found to be overexpressed in EOC samples and prevent apoptosis in EOC cells by repressing the inflammasomes [91]. In EOC patients, Fra-2 levels do not correlate with any histological features like subtype, Grading or FIGO staging [92]. More recently, it has been found that Fra-2 expression is modulated by miR-143 and significantly correlates with drug response in EOC samples from platinum treated patients [93]. In this setting, upregulation of LncRNA-UCA1 determines Fra-2 overexpression by sponging miR-143, eventually leading to the platinum resistance in EOC [93].

### Role of Fra-2 in high grade gliomas

High-grade gliomas (HGG) are the most common brain tumors in humans and are essentially incurable. Gene expression studies have established that over-expression of a mesenchymal gene expression signature (MGES) identifies a subgroup of patients with a more aggressive disease and poor prognosis. Importantly, Fra-2 represents one of the six transcription factors that collectively controls about 75% of the MGES genes in HGG [94]. In chromatin immunoprecipitation validation experiment, Fra-2 binds and likely activates the promoter region of 93% of the MGES tested targets in HGG cells, contributing to tumor aggressiveness [94].

Fra-2 expression is also significantly associated with the so-called natural evolution signature (NES) of HGG, that encompasses a number of genes activated during the tumor progression [95]. According to this model, Fra-2 is expressed and activated in response to hypoxic stress and, by inducing NES, strongly contributes to the evolution of HGG. Moreover, Fra-2 transcriptionally regulates ANXA1 levels in HGG cells, which subsequently recruit monocytes in the TME, eventually promoting their inactivation (M2-phenotype) and immunosuppression [95].

### Role of Fra-2 in T-cell malignancies

In literature, Fra-2 is reported to play a critical role in lymphocytes.

Fra-2 is a key upstream regulator of Foxo1 and Irf4 expression that mediate proliferation, survival and rearrangements of both heavy and light chains in B-cells [96]. Therefore, Fra-2 regulates B cell development, and its deletion leads to an impairment of B cell maturation in the bone marrow [96].

In T cells, Fra-2 exerts a multifaceted role in controlling T helper (Th) lineage plasticity. Indeed, Fra-2 can repress Th17 and Th1 signature genes, controlling inflammatory response and preventing Th1 activation. By contrast, Fra-2 can also activate the transcriptomic program of Th17 cells, supporting their diversification and survival [97]. In thymocytes, TCR stimulation activates Fra-2 expression that, in turn, represses the expression of T regulatory (Treg)-specific genes like FoxP3 during the development of T-regulatory cells [98]. Therefore, Fra-2 overexpressing mice display a reduction of Treg and multi-organ autoimmunity [98].

**Fig. 5:**
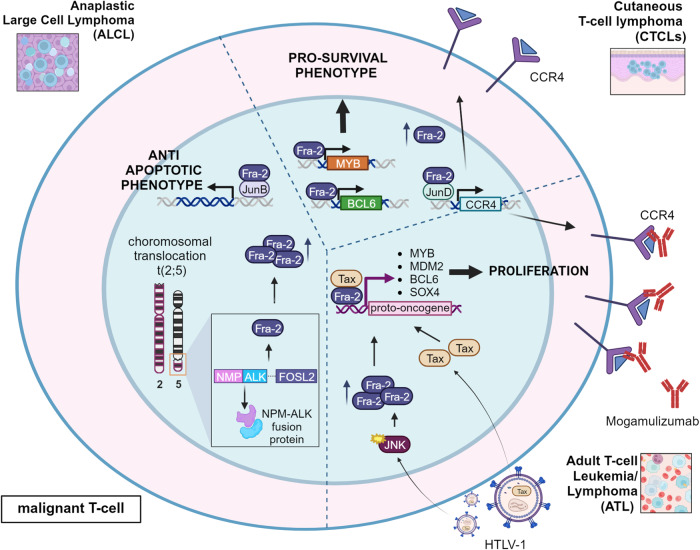
Role of Fra-2 in T-cell malignancies. In cutaneous T-cell lymphomas (CTCLs), Fra-2 overexpression transcriptionally activates CCR4, MYB and BCL6 proto-oncogenes. Even in adult T-cell leukemia/lymphoma (ATL), Fra-2 upregulation has a pro-survival role in transformed T-cells. HTLV-1 infection activates JNK that in turn induces Fra-2. The interaction between Tax and AP-1 complex, here represented by Fra-2, promotes the transcription of a various number of oncogenes (MYB, MDM2, BCL6, SOX4), contributing to cell growth and proliferation. Administration of mogamulizumab, an anti-CCR4 antibody, improves the patient outcome in relapsed or refractory ATL. In anaplastic large cell lymphoma (ALCL), characterized by the translocation t(2;5) and the constitutive activation of the NPM-ALK fusion protein, Fra-2 is also overexpressed. AP-1 complex, made of Fra-2 and Jun, inhibits the apoptotic pathway. Created with BioRender.

Importantly, Fra-2 expression has been related to the pathogenesis of different T-cell malignancies (adult T-cell leukemia, cutaneous T-cell lymphomas and anaplastic large cell lymphoma) (Fig. 5) and to the modulation of T-cell response in the TME, as discussed in the specific section.

Adult T-cell leukemia/lymphoma (ATL) is an aggressive lymphoproliferative neoplasm of mature CD4 + T-cells, etiologically associated with human T-lymphotropic virus type 1 (HTLV-1) infection [99]. In fact, HTLV-1 infection confers resistance to apoptosis and proliferative properties to infected cells, whereas other genomic events determine the transformation of T-cells (for instance, STAT3 and CCR4 genes), probably justifying the long latency from HTLV-1 infection to ATL onset [100]. The immortalizing entity of HTLV-1 has been attributed to the viral transcriptional trans-activator protein Tax [101].

To evade apoptosis and commence proliferation, Tax activates the transcription of several cellular genes that are implicated in cytokines signaling, T-cell receptor-NF-kB pathway, T-cell trafficking and immunosurveillance [100]. Importantly, Tax is incapable of activating gene promoters by itself; therefore, the interaction between Tax and enhancer elements is mediated by recruiting cellular transcription factors and modulators, mainly represented by NF-kB, E2F and AP-1 [12, 102, 103]. In T-cell lines, HTLV-1 infection leads to upregulation of AP-1 proteins, that are constitutively overexpressed in leukemic cells of ATL patients [103]. The AP-1 activation is supposed to be mediated by the stress activated protein kinase JNK, which is constitutively activated following HTLV-1 infection.

Consistently, primary resting CD4 + T-cells were negative for Fra-2, whereas ATL cells showed a strong upregulation of Fra-2 [104].

In this context, Fra-2 plays a critical role in the leukemogenesis of T-cells [104]. Gene expression analysis of control and Fra-2 silenced ATL cells showed that Fra-2 promotes the upregulation of notable proto-oncogenes like c-Myb, MDM2, BCL6 and the transcription factor SOX4 [104, 105].

By c-Myb, MDM2 and BCL6 upregulation, Fra-2 contributes to the pro-survival phenotype of transformed T-cells, whereas Fra-2 and SOX4 forms an important oncogenic cascade leading to the transcription of several other genes involved in ATL cell growth [105]. Consistently, Fra-2 silencing significantly reduced cell proliferation in ATL cell lines [104].

Moreover, Fra-2/JunD dimers transcriptionally regulate the expression of CCR4 [104], a chemokine receptor strongly upregulated in the majority of ATL cells and found to be mutated in a significant proportion of ATL patients [100]. This role of Fra-2 in ATL could also have profound clinical implications, since mogamulizumab, an anti-CCR4 antibody, has been demonstrated to improve clinical response rate in relapsed or refractory ATL [99]. Therefore, it is conceivable that Fra-2 activation may sensitize ATL patients to mogamulizumab via CCR4 overexpression.

Importantly, the regulation of CCR4 by Fra-2 transcriptional activity has been also documented in cutaneous T-cell lymphomas (CTCLs), a heterogeneous group of mature T-cell malignancies that are not associated with HTLV-1 infection. Accordingly, Fra-2 results overexpressed in CTCLs human samples compared to normal skin and atopic dermatitis samples [106]. Consistently with ATL, also in CTCL cell lines Fra-2 silencing downmodulated expression of CCR4, MYB and BCL6, suggesting that the expression of those genes is controlled by Fra-2 [106].

Finally, Fra-2 is also overexpressed in anaplastic large cell lymphoma (ALCL), a subgroup of peripheral T cell lymphomas characterized by the translocation t(2;5). This translocation involves the 3’ anaplastic lymphoma kinase (ALK) tyrosine kinase domain located on 2p23, resulting in a constitutive activation of the NPM-ALK fusion protein [107]. Importantly, several genes surrounding the breakpoint regions on chromosomes 5 and 2 appear to be dysregulated in ALCL, encompassing FOSL2 which is located in spatial proximity to ALK gene (2p23) [107]. Immunohistochemical analysis of tissue sections ALCL patient samples harboring or not t(2;5) alteration revealed that Fra-2 expression is high in all translocation-positive ALCL cases and in a consistent number of translocation-negative ALCL cases [107]. These results lead to the intriguing speculation that Fra-2 dysregulation could anticipate and increase the potential for a chromosomal translocation [107].

As a result of this, ALCL cells showed increased levels of Fra-2, and Fra-2/JunB exerted an anti-apoptotic role in neoplastic lymphocytes [107].

### Role of Fra-2 in the tumor microenvironment

The tumor microenvironment (TME) encompasses all the cellular and acellular components located in close proximity to cancer cells which greatly contribute to the tumor initiation and evolution [108]. Neoplastic cells establish an active crosstalk with the non-neoplastic cells training the TME in a pro-tumorigenic manner [108].

**Fig. 6:**
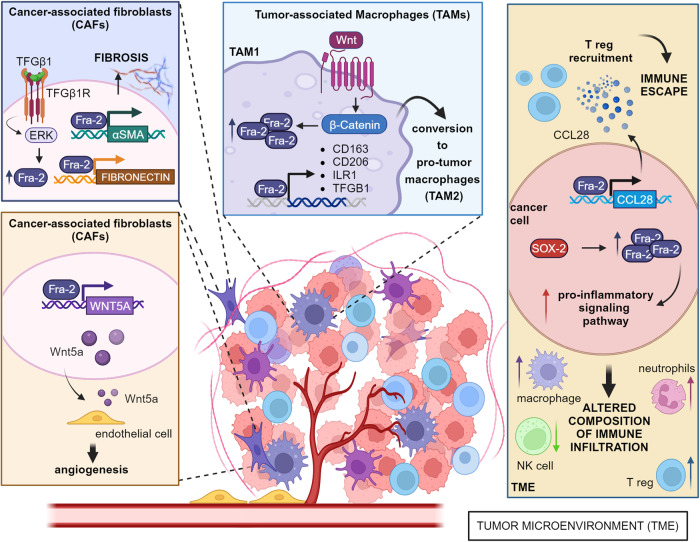
Role of Fra-2 in tumor microenvironment. In primary human breast fibroblasts, the activation of TGFβ1 signaling pathway engages Fra-2 expression *via* ERK and promotes Fibronectin and αSMA. This mechanism contributes to tumor microenvironment remodeling and tumor progression in breast cancer. In breast cancer-associated fibroblasts (CAFs), Fra-2 transcriptionally activates WNT5A, stimulating endothelial cells and neo-angiogenesis. In tumor-associated macrophages (TAMs), Wnt/β-catenin signaling induces Fra-2 activation that in turn promotes transcription of CD163, Cd206, ILr1 and TGFB1, supporting the switch from TAM1 to pro-tumorigenic macrophages (TAM2). In PDAC, Fra-2 overexpression transcriptionally activates CCL28, recruiting Treg cells. In a SOX2 amplification context, linked to tumor aggressiveness, Fra-2 activation is enhanced. Both these mechanisms eventually promote immune escape, with an increased number of Treg, macrophages and neutrophils and a decrease of activated natural killer (NK) cells. Created with BioRender.

In this context, Fra-2 expression has been observed in both cancer and cancer-associated cells, playing a crucial role in TME reshaping (Fig. 6). As previously discussed, PDAC is associated with an extremely unfavorable TME, where hypoxic stress and starvation are strong inducers of Fra-2 upregulation in cancer cells [10, 75]. Fra-2-espressing PDAC cells activate transcription and secretion of C-C motif chemokine ligand 28 (CCL28), a chemokine involved in the recruitment of regulatory T cells, eventually promoting a suppressive immune microenvironment [74].

As previously discussed, Fra-2 upregulation favors proliferation, migration and metastatic dissemination of CRC cells, eventually promoting tumor progression [7, 79, 80].

However, when the interplay between neoplastic and non-neoplastic cells is considered, the significance of Fra-2 expression drastically changes, since it acts as a critical mediator of sensitivity to therapeutic administration. Indeed, it has been reported that Fra-2 levels positively correlates with CD8 + T cell infiltration and tumor regression in rectal cancer samples from patients treated with chemo-radiotherapy [109]. Mechanistically, the therapy-induced overexpression of Fra-2 increases CXCL10 secretion and CD8 + T cell recruitment in the TME, enhancing the cytotoxic response against CRC cells [109].

A pan-cancer in-silico study revealed that SOX2 amplification is strictly associated with tumor aggressiveness and activates Fra-2 expression. In turn, Fra-2 mediates the SOX2-induced upregulation of several pro-inflammatory signaling pathways that alter the composition of the immune infiltration in the TME. In PDAC samples, prolonged inflammation significantly increases Tregs, macrophages and neutrophils, and decreases activated NK cells, eventually evolving into immunosuppression [110].

Tumor-associated macrophages (TAMs) represent a heterogeneous population of the immune niche comprehending activated, proinflammatory, anti-tumor cells (called tumor inhibiting macrophages, TAM1) and regulatory, anti-inflammatory cells (pro-tumor macrophages, TAM2). The M2-phenotype correlates with tumor progression and limits the efficacy of immune checkpoint inhibitors. In preclinical models of NSCLC, Wnt/β-catenin signaling induces Fra-2 activation and ARID5A repression in macrophages, driving the expression of M2-specific genes (CD163, CD206, IL1R1 and TGFβ) and leading to the differentiation toward M2-phenotype [111]. Interestingly, pharmacological inhibition of β-catenin binding to the promoter regions of both Fra-2 and ARID5A, reverts the phenotype switch of TAM2 to TAM1, eventually decreasing NSCLC growth and metastasis [111]. Altogether, these results indicate that β-catenin–mediated activation of Fra-2 induces immune escape of NSCLC by triggering the pro-tumorigenic transcriptional program of M2-like macrophages.

Cancer-associated fibroblasts (CAFs) represent a major component of TME, contributing to ECM remodeling, angiogenesis and tumor resistance to therapeutics. Compared to normal fibroblasts, CAFs exhibit drastic changes at transcriptomic level, driven by the activation of several transcription factors, including Fra-2. In BC-associated fibroblasts, Fra-2 expression significantly correlates with the vascular density of tissue samples [112]. Mechanistically, Fra-2 regulates the transcription of Wnt5a in CAF, a secreted glycoprotein, that induces neo-angiogenesis *via* FZD5/NF-kB/ERK signaling in the endothelial cells [112].

## Conclusions and opportunities

The transcription factor Fra-2 is ubiquitously expressed in body cells and is deeply associated with homeostasis and differentiation of tissues. Accumulated evidence points to a critical involvement of Fra-2 in different human diseases and, particularly, in cancer as we summarized in the above sections.

Except for anaplastic large cell lymphoma [107], in which ALK rearrangements have been linked with alteration of FOSL2, there are no evidence of mutational events involving FOSL2 gene that drive human malignancies. Consistently, Fra-2 transgenic mice display a fibrotic disease reminiscent of systemic sclerosis without developing spontaneous tumors [27, 32, 33].

Therefore, the upregulation of Fra-2, reported in several cancer types, represents the ancillary consequence of other mechanisms like dysregulation of microRNAs, mutations in proto-oncogenes and aberrant activation of signaling cascades. However, Fra-2 induction does not represent a minor event, since it is the mediator between oncogenic stimuli and transcriptional rewiring. The result of this intricate crosstalk is not so obvious: indeed, Fra-2 may act as an oncosupressor (such as in MM) [18] or as an oncogene (like in BC) [17, 53]. Intriguingly, the effect of Fra-2 activity varies not only in a tumor-dependent but also in a context-dependent manner. In the late stages of cancer progression Fra-2 critically sustains the evolution of CRC, NSCLC and EOC, while in the early stages Fra-2 works differently, by counteracting or being dispensable for carcinogenesis.

This discrepancy likely relies on the capability of Fra-2 to promote migration, invasion and metastatic dissemination that are key features of advanced tumors. In this regard, Fra-2 is closely interconnected with TGFβ pathway and directly activates EMT-promoting genes. Considering Fra-2 as an immediate early gene, its involvement in cell plasticity mechanisms seems to be reasonable. In fact, Fra-2 is rapidly induced by many stressors such as hypoxia [10], serum deprivation [75], immune response [111] and therapeutic administration [6, 93].

In this context, Fra-2 inhibition may be a valuable strategy to contrast cancer cell plasticity, immune escape and drug-resistance.

Recently, the introduction of AP-1 inhibitors [64] has opened the way to novel pharmacological opportunities to improve the efficacy of targeted [65, 66] and cytotoxic therapies [6]. For instance, since PDAC frequently harbors RAS mutation, is exposed to microenvironmental stress and immune pressure [72] and undergoes to EMT, it represents an ideal candidate for these drugs. It is still unexplored if these strategies could impact the other AP-1 proteins. Future investigations need to assess whether and how these therapeutic approaches could be translated in the clinical practice.
